# The Prevalence and Phenotype of Activated Microglia/Macrophages within the Spinal Cord of the Hyperostotic Mouse (*twy/twy*) Changes in Response to Chronic Progressive Spinal Cord Compression: Implications for Human Cervical Compressive Myelopathy

**DOI:** 10.1371/journal.pone.0064528

**Published:** 2013-05-24

**Authors:** Takayuki Hirai, Kenzo Uchida, Hideaki Nakajima, Alexander Rodriguez Guerrero, Naoto Takeura, Shuji Watanabe, Daisuke Sugita, Ai Yoshida, William E. B. Johnson, Hisatoshi Baba

**Affiliations:** 1 Department of Orthopaedics and Rehabilitation Medicine, Faculty of Medical Sciences, University of Fukui, Eiheiji, Fukui, Japan; 2 Life & Health Sciences, Aston University, Aston Triangle, Birmingham, United Kingdom; Virginia Commonwealth University, United States of America

## Abstract

**Background:**

Cervical compressive myelopathy, e.g. due to spondylosis or ossification of the posterior longitudinal ligament is a common cause of spinal cord dysfunction. Although human pathological studies have reported neuronal loss and demyelination in the chronically compressed spinal cord, little is known about the mechanisms involved. In particular, the neuroinflammatory processes that are thought to underlie the condition are poorly understood. The present study assessed the localized prevalence of activated M1 and M2 microglia/macrophages in *twy/twy* mice that develop spontaneous cervical spinal cord compression, as a model of human disease.

**Methods:**

Inflammatory cells and cytokines were assessed in compressed lesions of the spinal cords in 12-, 18- and 24-weeks old *twy*/*twy* mice by immunohistochemical, immunoblot and flow cytometric analysis. Computed tomography and standard histology confirmed a progressive spinal cord compression through the spontaneously development of an impinging calcified mass.

**Results:**

The prevalence of CD11b-positive cells, in the compressed spinal cord increased over time with a concurrent decrease in neurons. The CD11b-positive cell population was initially formed of arginase-1- and CD206-positive M2 microglia/macrophages, which later shifted towards iNOS- and CD16/32-positive M1 microglia/macrophages. There was a transient increase in levels of T helper 2 (Th2) cytokines at 18 weeks, whereas levels of Th1 cytokines as well as brain-derived neurotrophic factor (BDNF), nerve growth factor (NGF) and macrophage antigen (Mac) −2 progressively increased.

**Conclusions:**

Spinal cord compression was associated with a temporal M2 microglia/macrophage response, which may act as a possible repair or neuroprotective mechanism. However, the persistence of the neural insult also associated with persistent expression of Th1 cytokines and increased prevalence of activated M1 microglia/macrophages, which may lead to neuronal loss and demyelination despite the presence of neurotrophic factors. This understanding of the aetiopathology of chronic spinal cord compression is of importance in the development of new treatment targets in human disease.

## Introduction

Mechanical compression of the spinal cord can cause neural tissue damage, reduction of neuronal cell activity and protein synthesis, and neuronal cell death. Cervical compressive myelopathy is characterized by progressive stenosis of the cervical canal and compression of the spinal cord due to spondylosis, degenerative disc disease, and ossification of the posterior longitudinal ligament (OPLL) [1]–[4]. Symptoms usually start to appear after middle age with slowly progressive clumsiness and paresthesia in the hands, gait disturbance, and signs of posterior and pyramidal column dysfunction; eventually leading to tetraplegia or tetraparesis. Human pathological studies have reported flattening of the anterior horns, loss of anterior horn cells, cavity formation, ascending demyelination in the posterior columns, descending demyelination in the lateral columns and proliferation of hyalinized small blood vessels [5]–[7]. However, since it is difficult to properly estimate and follow the progression of these changes in humans, considerable uncertainty exists regarding the biological and molecular mechanisms responsible for the demyelination that takes place in these disorders, and for the progressive loss of neurons and oligodendrocytes. Moreover, although pro-inflammatory cytokines and related immune effector molecules are considered to be produced after chronic, slow compression of the spinal cord as seen in cervical compressive myelopathy, and could result in induction of cell death through necrosis and apoptosis, their role in compression-induced damage remain questionable [8], [9].

The inflammatory changes following spinal cord injury (SCI) are complex and involve the activation of resident microglia and recruitment of neutrophils, macrophages and lymphocytes into the lesion from the systemic circulation [10]–[12]. This leads to tissue damage, demyelination and neurological dysfunction [13], as well as apoptosis of neurons and oligodendrocytes [14], [15]. Activated microglia and recruited macrophages (which are antigenically not distinguishable, henceforth these cell types have been referred to as microglia/macrophages) are implicated in neuroinflammation through the induction or modulation of a broad spectrum of cellular responses [10]. These cells are the primary source for pro-inflammatory cytokines within the spinal cord, and their presence and activity have the potential to act as markers of disease onset and prognosis of neurological outcome following SCI [13], [16]. Interestingly such neuroinflammation, including the microglial response, has also been identified as a contributor to cell death in ischemic injury in the brain [17] and chronic neurodegenerative disorders [18]. These findings point to the potential commonality of mechanisms underlying cell damage and cell death in both acute neural injury and in slow-developing pathologies of neural systems, like those seen in Alzheimer’s disease and Parkinson disease, even in the absence of prominent leukocyte infiltration [18].

Recent studies have demonstrated phenotypic changes in macrophages during the immunological and inflammatory responses to various conditions [19], [20]. This divergence is referred to as macrophage polarization and has been reported in non-neural [21] and neural tissues [22], [23], and also in both *in vitro* and *in vivo* experiments [24]. Approximately, two subtypes of macrophages have become of great interest in the field of spinal cord regeneration: classically activated macrophages (M1 phenotype) and alternatively activated macrophages (M2 phenotype) [25]–[28]. Whereas the M1 phenotype is the product of exposure to T helper 1 (Th1) cytokines, such as interferon gamma (IFN-γ), tumor necrosis factor-alpha (TNF-α), and interleukin (IL)-6, the M2 phenotype is activated via T helper 2 (Th2) cytokines, such as IL-4, IL-10, and IL-13 [22], [29], [30]. While the M1 phenotype is known for their high expression of inflammatory cytokines and bactericidal activity, M2 phenotype exhibits enhanced phagocytic and anti-inflammatory properties; although at least three subsets of M2 macrophages have also been documented [21], [25]; for such reasons, macrophages with the latter phenotype are considered to function in recovery of SCI [19], [23], [31]. Thus, modification of the SCI microenvironment to increase the number of M2 macrophages may promote neuroprotection. A similar possibility has also been recently attributed to microglia by showing that these cells can also be induced under certain conditions to both extremes of the M1 and M2 differentiation spectrum [25], [32].

The study of the pathological mechanisms of spinal cord dysfunction related to cervical spondylotic myelopathy (CSM) and OPLL has been impaired in the past due to the lack of good *in vivo* models. However, this has changed with the recent characterization of the tip-toe Walking Yoshimura (*twy/twy*) mouse; an autosomal recessive mutant. The *twy/twy* mouse has a spontaneous mutation in the nucleotide pyrophosphatase (*Npps*) gene developing posterior calcification of the atlantoaxial membrane at the cervical (C) 1-C2 vertebral level. The defective vertebral column causes cervical spinal cord compression progressively over several months. Thus, our group has used the *twy/twy* mouse as a suitable model to investigate the effects of the chronic, slow compression of the spinal cord that is seen in CSM and OPLL [33]–[36].

Recent studies have suggested that neuronal and oligodendrocytic apoptosis through activation of the Fas death receptor pathway is a key event in the *twy/twy* mouse spinal cord [37] and have shown that neutralization of Fas ligand with a function-blocking antibody reduced neural inflammation at the lesion mediated by activated microglia and macrophages [7]. Another study from our laboratory reported that increased expression of TNF-α and TNF receptor 1 (TNFR1) released by the activated microglia/macrophages correlated with neuronal and oligodendrocytic apoptosis [8]. Based on the above findings, the present study was designed to provide insight on previously unexplored aspects of microglia/macrophage phenotypic changes induced by chronic, slow spinal cord compression seen in cervical compressive myelopathy. Specially, we investigated the expression and colocalization of markers of microglia/macrophages (both M1 and M2 phenotypes) as well as the levels of neuroinflammatory cytokines closely related to these cells, which could promote neurotoxicity or neuroprotection and lesion repair in the *twy/twy* spinal cord.

## Materials and Methods

### The Spinal Hyperostotic *twy*/*twy* and Control Mice and Spinal Cord Progressive Compression Evaluated by Computed Tomography (CT)

The Ethics Review Committee for Animal Experimentation of University of Fukui approved the experimental protocol. Spinal hyperostotic *twy/twy* mice purchased from the Central Institute for Experimental Animals (Kawasaki, Japan), were used in all experiments (aged 12 weeks; n = 29, 18 weeks; n = 29, 24 weeks; n = 29) (Table S1). Homozygous *twy/twy* mice were maintained by brother-sister mating of heterozygous Institute of Cancer Research (ICR) mice (*+/twy*). ICR mice at the age of 12, 18, and 24 weeks were used as control animals (n = 10 in each age group). The disorder is inherited in an autosomal recessive manner and the homozygous hyperostotic mouse is identified by a characteristic tip-toe walking at 6 to 8 weeks of age, but no congenital neurological abnormalities are detected at that age. The *twy/twy* mouse exhibits spontaneous calcified deposits posteriorly at the C1–C2 vertebral level, producing a variable degree of compression of the spinal cord between C2 and C3 cord segments with a general ankylosis of joints. The calcified mass grows in size progressively with age particularly in the atlantoaxial membrane, causing profound motor paresis at the age of 18–24 weeks [9], [33], [37].

For hematoxylin and eosin (H&E) staining, the resected cervical spine of each *twy*/*twy* mouse of different ages (n = 5 for each time point) was fixed in buffered formaldehyde for 48 hours at 4°C. The sample was then decalcified for 2 weeks at 4°C in 0.5 M ethylenediaminetetraacetic acid (0.5 M Tris-HCl buffer) at pH 7.6 and then embedded in paraffin using standard procedures. Serial 4-µm-thick cryostat sagittal and axial sections were prepared.

In order to confirm differences in the severity of compression before H&E staining, flow cytometry, and immunoblot analysis, we measured the spinal canal area in the cervical spine of *twy*/*twy* mice on CT scans (GE Medical Systems, Milwaukee, WI) obtained under anesthesia with ravonal (Tiopental^®^, Mitsubishi Tanabe Pharma, Osaka, Japan), using Image J, the image analysis software of the National Institutes of Health (Bethesda, MD). The correlation between age and spinal canal area at the site of maximum compression at C1–C2 vertebral level was determined. We also compared the spinal canal areas at C1–C2 to that at thoracic (Th) 1 vertebral level. In the same way, we also compared the spinal cord areas at the site of maximum compression in H&E staining at C1–C2 to that at Th1 vertebral level using the color image analyzer (MacSCOPE, Minani, Fukui, Japan).

### Immunohistochemistry

Deep anesthesia was induced in each group of mice (n = 5 *twy*/*twy* mice and n = 2 control ICR mice for axial sections, and n = 2 *twy*/*twy* mice for sagittal sections in each time point) followed by transcardial perfusion and fixation with 4% paraformaldehyde in 0.1 M phosphate-buffered saline (PBS); the spinal cords were dissected and post-fixed in the same fixative for a few hours. The tissue samples were immersed in 10% sucrose in 0.1 M PBS at 4°C for 24 hours, and 30% sucrose in 0.1 M PBS for 24 hours. Segments of the cervical spinal cord were embedded in optimal cutting temperature compound (Sakura Finetek, Torrance, CA) and cut on a cryostat into serial 10 µm-thick axial or sagittal frozen sections, which were serially mounted on glass slides and fixed with 2% paraformaldehyde in 0.1M PBS for 5 minutes, rinsed in PBS and stored at −80°C.

For immunofluorescence staining, frozen sections were permeabilized with 0.1 M Tris-HCl buffer (pH 7.6) containing 0.3% Triton X-100. The following primary antibodies diluted in Antibody Diluent with Background Reducing Components (Dako Cytomation, Carpenteria, CA) were applied overnight at 4°C: rabbit anti-Integrin αM (equivalent to CD11b), 1∶200 (Santa Cruz Biotechnology, Santa Cruz, CA); mouse anti-neuronal nuclei (NeuN) monoclonal antibody, 1∶400 (Millipore Corporation, Billerica, MA); rabbit anti-inducible nitric oxide synthase (iNOS), 1∶200 (BD Pharmingen, San Jose, CA); rat anti-CD16/32, 1∶200 (Santa Cruz Biotechnology); goat anti-arginase-1, 1∶200 (Santa Cruz Biotechnology); goat anti-CD206, 1∶200 (Santa Cruz Biotechnology); rabbit anti-brain derived neurotrophic factor (BDNF) polyclonal antibody, 1∶300 (Abcam plc, Cambridge, UK); rabbit anti-nerve growth factor (NGF) polyclonal antibody, 1∶300 (Abcam plc); anti-macrophage antigen-2 (Mac-2), 1∶200 (BioLegend, San Diego, CA); and mouse monoclonal anti-CD4 antibody, 1∶100 (Abcam plc). The sections were then incubated for 1 hour at room temperature with Alexa Fluor-conjugated 488- or 568- secondary antibodies, 1∶250 (Molecular Probes, Eugene, OR). Finally, the sections were washed, wet-mounted, and examined by the omission of a primary antibody or through the use of a non-specific negative primary antibody that was isotype matched. Furthermore, some sections were counterstained with nuclear marker DAPI (Abbott Molecular, Des Plaines, IL).

All images were obtained using a fluorescence microscope (Olympus AX80, Olympus Optical, Tokyo) or a confocal laser scanning microscope (model TCS SP2, Leica Instruments, Nussloch, Germany), where the 488- and 543-nm lines of the argon/helium-neon laser were used for fluorescence excitation.

### Semi-quantitative Analysis of Stained Tissues

Changes in CD11b-(red), NeuN- (green) and CD4- (red) positive areas at 12, 18, and 24 weeks of age *twy*/*twy* mice, and control ICR mice were assessed by the following procedure: serial axial sections were divided into five groups (slide glass) by collecting every fifth section separately from the site of maximum compression (between the C2 and C3 dorsal roots) and half of the spinal cord on the compressed side was analyzed using grain counting with the light intensity automatically set by the color image analyzer (MacSCOPE).

The proportions of CD11b-positive cells double immunostained with iNOS, CD16/32, arginase-1 or CD206 in each groups were determined semi-quantitatively by the following procedure: the serial axial sections divided into five groups (slides) as mentioned above from the site of maximum compression (between the C2 and C3 dorsal roots) and the number of positive cells per cross-section in each fluorescence stain was determined automatically using grain counting based on light intensity by a color image analyzer (MacSCOPE). The light intensity and threshold values were maintained at constant levels when collecting digitized images in all analysis. We documented the extent to which the microglia/macrophages present within the spinal cord were polarized by the M1/M2 ratio, as determined by the number of CD11b cells that were also positive iNOS and CD16/32/the number of CD11b cells that were also positive for CD206 and arginase-1.

### Flow Cytometry

Immediately after deep anesthesia, the mouse was perfused intracardially with 200 ml of ice-cold 0.1 M PBS, and the spinal cords were harvested (n = 3 for each time point). The cervical spinal cord around the maximally compressed site was surgically dissected and dissociated with collagenase, 175 U/ml (Sigma-Aldrich, St. Louis, MO) for 1 hour at 37°C. Cells were washed in Dulbecco’s modified Eagle’s Medium (Invitrogen Life Technologies, Carlsbad, CA) containing 10% fetal bovine serum and filtered through a 40 µM nylon cell strainer (BD Biosciences, San Jose, CA) under centrifugation to remove tissue debris and obtain a single-cell suspension, as described in detail previously [38].

From this point on, prior to every staining, a cell-count was performed in every sample to ensure a cell density of 1.0×10^6^ cells/100 µL. Cells were incubated for 1 hour on ice with the following fluorescent antibodies: allophycocyanin (APC) rat anti-CD45, 0.25 µg/ml (BioLegend, San Diego, CA); Pacific Blue rat anti-Ly-6G/Ly-6C, 1.0 µg/ml (equivalent to Gr-1; BioLegend) and PerCP-Cy 5.5 rat anti-CD11b, 0.25 µg/ml (BD Pharmigen, San Jose, CA). For intracellular staining [39], the cells were resuspended in Fixation buffer and treated with Permeabilization Buffer (both from Santa Cruz Biotechnology) followed by re-suspension in ice-cold PBS and incubation for 1 hour with goat anti-arginase 1, 1∶200 conjugated to fluorescein isothiocyanate (FITC),1∶200 (Santa Cruz Biotechnology) and phycoerythrin (PE)-conjugated rabbit anti-iNOS, 3.0 µg/ml (Abcam plc); or PE/Cy7 conjugated rat anti-CD16/32, 1.0 µg/ml (Biolegend) and FITC rat anti-CD206, 1.0 µg/ml (Biolegend). Samples with cells alone were used as negative controls to eliminate background autofluorescence, and samples where the cells had been incubated with a single-added antibody were used as positive controls to set up the cytometer alignment and to remove any spectral overlap.

Flow cytometry was performed immediately using a FACS Canto™ II (Becton Dickinson Biosciences, San Jose, CA). Forward scatter was set to further eliminate any cellular debris from analysis. In each test, a minimum of 250,000 cells were analyzed and the data were processed using BD FACSDiva software (Becton Dickinson Biosciences). The different cell populations present in the suspension were classified according to the combination of expressed antigens, as stated in previous reports, as follows: CD11b^high^/CD45^low^/GR-1^negative^ represented resting microglia [39], and CD11b^high^/CD45^high^/GR-1^negative^ represented activated microglia/macrophages [38]. At the previously described time points, CD11b^high^ cells in the spinal cord were sub-fractioned into a CD45^low^/GR-1^negative^ population, identifying them as resting microglia. In a similar fashion, CD11b^high^ cells were sub-fractioned into a CD45^high^/GR-1^negative^ population, which identified them as activated microglia/macrophages.

The phenotype of microglia/macrophage sub-populations was corroborated through their expression of iNOS or CD16/32 (pro-inflammatory M1 phenotypes) as well as arginase 1 and CD206 (anti-inflammatory M2 phenotypes).

### Myeloperoxidase (MPO) Staining and Assay

The 3,3′-diaminobenzidine (DAB) staining kit (Muto Pure Chemicals Co., Tokyo) was used in each mouse (n = 3 for each time point) for cytochemical staining of MPO according to the instructions supplied by the manufacturer. Briefly, the peroxidase reaction was developed with 0.05% 3,3′-DAB in 50 mmol/L Tris-HCl (pH 7.6) and 0.03% H_2_O_2_ for 1 to 2.5 min. The sections were counterstained with eosin, dehydrated, and mounted. Oxidized 3,3′- DAB (a brown, highly insoluble indamine polymer) was visible under light microscopy.

MPO activity levels in harvested compressed spinal cord tissues (n = 3 for each time point) were measured with MPO assay kit (BioVision, Milpitas, CA) using a spectrophotometer at 412 nm. One unit of MPO activity was defined as the amount of enzyme degrading 1 µmol of 5-thio-2-nitrobenzoic acid (TNB) per minute at 25°C. MPO activities in the spinal cord tissues were calculated by using a standard curve generated with MPO and expressed in units per miligram weight of wet tissue.

### Immunoblot Analysis

Immediately after deep anesthesia, the spinal cord of each mouse (n = 3 for each time point) around the maximally compressed site (between C2 and C3 dorsal roots) was carefully dissected *en bloc* from the cervical spine and stored immediately at −80°C in liquid nitrogen. Segments were centrifuged at 15,000×*g* for 30 seconds using a BioMasher Rapid Homogenization Kit (Funakoshi, Tokyo), then solubilized in RIPA lysis buffer 1X (Santa Cruz Biotechnology), homogenized and stored at −80°C. The protein concentration was determined in the obtained samples by a Lowry protein Assay using a DC protein assay kit (Bio-Rad Laboratories, Hercules, CA). Laemmli sodium dodecylsulfate buffer samples containing the protein mixtures were boiled and subjected to immunoblot analysis. Total protein (20 µg/lane) was separated on 12.5% SDS-PAGE and transferred onto polyvinylidene difluoride membrane (PE Applied Biosystems, Foster, CA) for 70 minutes using a semi-dry blot apparatus. The membrane was washed twice in PBS containing 0.05% Tween 20, blocked by 5% skimmed milk in PBS for 1 hour at room temperature, and then incubated with one of the following antibodies: rabbit anti- IFN-γ, 0.2 µg/ml (Abcam plc), rabbit anti- TNF-α, 0.2 µg/ml (Abcam plc), rabbit anti- IL-6, 1∶200 (Santa Cruz Biotechnology), rat anti-IL-4, 1∶200 (Santa Cruz Biotechnology), rabbit anti-IL-10, 1∶200 (Santa Cruz Biotechnology), goat anti-IL-13, 1∶200 (Santa Cruz Biotechnology), rabbit anti-BDNF, 1∶200 (Abcam plc), rabbit anti-NGF, 1∶200 (Abcam plc), or rat anti-Mac-2, 1∶200 (BioLegend) overnight at 4°C. After triple washing in 0.1 M PBS, the membranes were incubated for 1 hour in the respective secondary IgG/HRP complex antibodies: anti-goat, 1∶1,000; anti-rabbit, 1∶5,000; or anti-rat, 1∶1,000 (all from Santa Cruz Biotechnology). After triple washing with 0.1 M PBS, the membrane was immersed in ECL Advance Western Blot Detection kit (GE Healthcare, Buckinghamshire, UK) for 1 minute and then exposed to X-ray film for visualization of peroxidase activity and determination of the level of each specific protein. The band intensities were normalized to β-actin, 1∶2000 (Abcam plc), and Kaleidoscope Prestained Standards (Bio-Rad Laboratories) was used as the molecular weight control.

### Statistical Analysis

All values are expressed as mean± standard deviation (SD). Differences between groups were examined for statistical significance using one-way factorial analysis of variance (ANOVA). Before the priori comparison, Kolmogorov-Smirnov test was used for verification of normality. A p value <0.05 denoted the presence of a significant difference with Tukey’s post hoc analysis. The above tests were conducted using SPSS software version 11.0 (SPSS, Chicago, IL).

## Results

### Chronic and Slow Progressive Compression in *twy*/*twy* Mouse Induces the Increase of Activated Microglia/Macrophages

**Figure 1:**
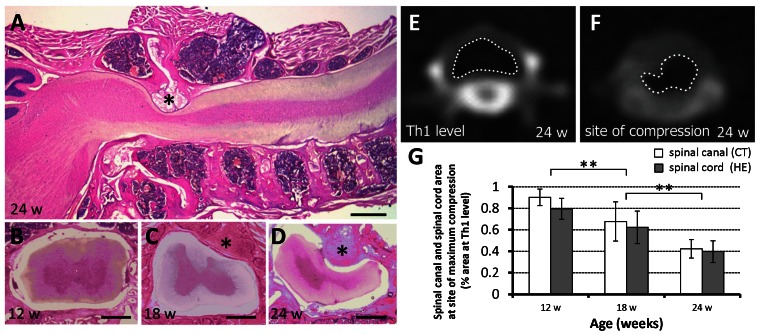
Histological and imaging evidence of progressive cervical spinal compression in *twy/twy* mice. Microphotographs of hematoxylin and eosin (H&E)-stained sagittal (A) and transaxial (B–D) sections, and computed tomography (CT) scans (E, F) of the cervical spine of 12- (B), 18- (C) and 24-week-old *twy/twy* mice (A, D, E, F). Calcified lesions originating from the atlantoaxial membrane increased in size progressively with age, compressing the lateral and dorsal aspects of the spinal cord between C2 and C3 segments (*) calcified lesions. A spinal canal transverse area on CT at thoracic (Th) 1 level and the site of compression of a 24-week-old *twy/twy* mouse is surrounded by white dotted line in (E, F). The relative spinal canal and spinal cord transverse area was shown compared with that of Th1 vertebral level assessed by CT and H&E staining (G). The spinal canal and spinal cord transverse area decreased with advancing age. Scale bars = 500 µm (A); 200 µm (B–D). **p<0.01 (n = 3 for each time point).

The *twy*/*twy* mouse exhibited a clear age-related compression of the spinal cord at the C1–C2 vertebral level. The calcified mass grew progressively with age particularly in the atlantoaxial membrane posteriorly at the C1–C2 vertebral level (Figs. 1A–D). CT scans and H&E staining demonstrated that the spinal canal and spinal cord transverse area rate at the C1–C2 relative to that at the Th1 vertebral level was 0.902±0.077 and 0.795±0.098 in 12-week-old, 0.677±0.162 and 0.623±0.151 in 18-week-old, 0.423±0.107 and 0.397±0.101 in 24-week-old *twy*/*twy* mice, respectively (Figs. 1B–G). These results indicated there is correlation between the CT findings and histological examinations, and that the spinal canal and spinal cord transverse area decreased with advancing age.

**Figure 2:**
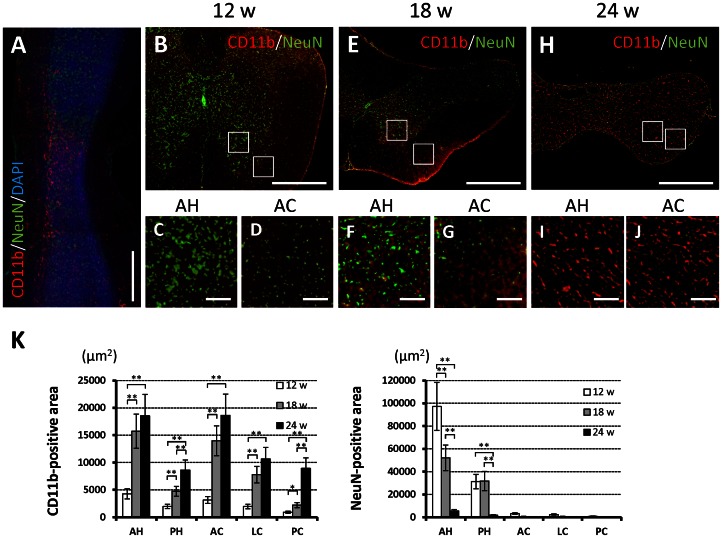
Increased prevalence of activated microglia/macrophages after spinal cord compression correlates to neuronal changes in *twy/twy* mice. Immunofluorescence staining for the expression of CD11b (red) and NeuN (green) in 12- (B–C), 18- (A, E–G) and 24-week-old (H–J) *twy/twy* mice. In sagittal sections of the spinal cords of 18-week-old *twy/twy* mice, CD11b-positive cells were distributed mainly in the sites of maximal compression site, compared with those sites that were rostral or caudal to the cord compression (A). The CD11b-positive area increased with the worsening of spinal cord compression, both in the gray and white matter, especially in the anterior horn (C, F, I) and anterior/lateral column (D, G, J). The NeuN-positive area of mainly the gray matter was especially lower in 24-week-old *twy/twy* mice (K). Scale bars = 500 µm (A, B, E, H); 50 µm (C, D, F, G, I, J). *p<0.05, **p<0.01 (n = 5 for each time point). AH: anterior horn, PH: posterior horn, AC: anterior column, LC: lateral column, PC: posterior column. A–J microphotographs were taken using confocal laser scanning microscope.

The area of the spinal cord that was positive for CD11b immunostained increased and that area which was positive for NeuN immunostained cells decreased with advancement of spinal cord compression. The CD11b-positive area increased according to the degree of spinal cord compression in both the gray and white matters, especially in the anterior horn and anterior column of the maximally compressed site compared with the rostral or caudal sites of the spinal cord (Fig. 2A). On the other hand, the NeuN-positive area, mainly in the anterior horn, decreased accordingly to the degree of spinal cord compression; the difference between 18- and 24-week-old *twy*/*twy* mice was significant (Fig. 2). The results of ICR mice in each age group (12-, 18-, and 24-week-old) were the same as those from 12-week-old *twy*/*twy* mice (Fig. S1A).

### Chronic Progressive Spinal Cord Compression Induces Changes in the Phenotype Markers of Microglia/Macrophages

**Figure 3:**
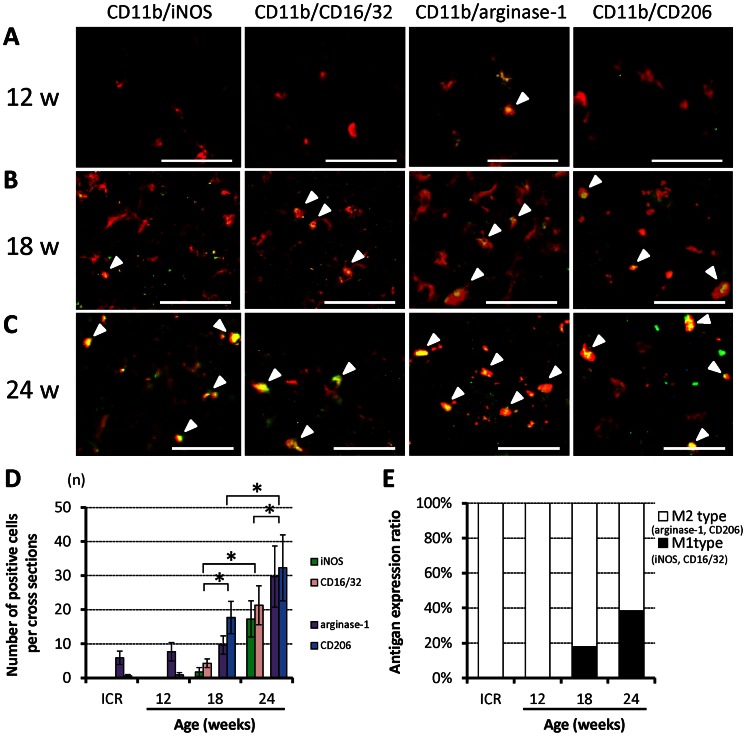
The prevalence of phenotypically activated microglia/macrophages in association with increased severity of spinal cord compression in *twy/twy* mice. Immunofluorescence staining for the expression of iNOS and CD16/32 (green) for classically activated microglia/macrophages (M1 phenotype) and arginase-1 and CD206 (green) for alternatively activated microglia/macrophages (M2 phenotype) co-localized with CD11b (red) in the anterior column of 12- (A), 18- (B) and 24-week-old (C) *twy/twy* mice. The numbers of CD11b-, CD11b/iNOS- and CD11b/CD16/32-positive cells (arrow heads) increased with the worsening of spinal cord compression. The CD11b/arginase-1- and CD11b/CD206-expressing cells (arrow heads) were the predominant population. The differences between iNOS, CD16/32 and arginase-1, CD206 were statistically significant in 18- and 24-week-old *twy*/*twy* mice. In control ICR mice, the expression of these factors was same as in 12-week-old *twy*/*twy* mice (D). The M1/M2 antigen expression ratio was higher in 24-week *twy*/*twy* mice compared with younger mice and control mice (E). Scale bars = 50 µm (A–C). Data are mean±SD. *p<0.05 (n = 5 for each time point). A–C microphotographs were taken using confocal laser scanning microscope.

To evaluate the phenotype of microglia/macrophages, tissues were immunostained with iNOS and CD16/32 for the M1 phenotype, and arginase-1 and CD206 for the M2 phenotype, as well as the pan-specific marker CD11b for microglia/macrophages. Double-positive merged cells were found particularly in the anterior horn and anterior column. In control ICR mice (with the same results seen in each age group; Fig. S1B) and in 12-week *twy*/*twy* mice, multiple cells co-expressing arginase-1 and CD11b were found; but no CD11b-positive cells co-expressing iNOS, CD16/32 or CD206 were identified (Fig. 3A). In 18-week *twy*/*twy* mice, while some CD11b-positive cells co-expressed iNOS or CD16/32, the number of those co-expressing arginase-1 or CD206 and CD11b remained higher (Fig. 3B). In 24-week *twy*/*twy* mice, while the number of CD11b- positive cells co-expressing iNOS or CD16/32 remained elevated, that of cells co-expressing CD11b and arginase-1 or CD206 persisted as the most abundant type (Fig. 3C). These differences between iNOS-CD16/32 and arginase-1-CD206 were statistically significant in 18- and 24-week-old *twy*/*twy* mice (Fig. 3D). Figure 3E shows the antigen expression ratio of M1 phenotype (CD11b-positive cells co-expressing iNOS or CD16/32)/M2 phenotype (CD11b-positive cells co-expressing arginase-1 or CD206) using immunofluorescence staining. The percentage of microglia/macrophages that was of M2 phenotype was 82.0% in 18-week-old and 61.6% in 24-week-old *twy/twy* mice with a concomitant increase in the percentage of microglia/macrophages that were of M1 phenotype.

**Figure 4:**
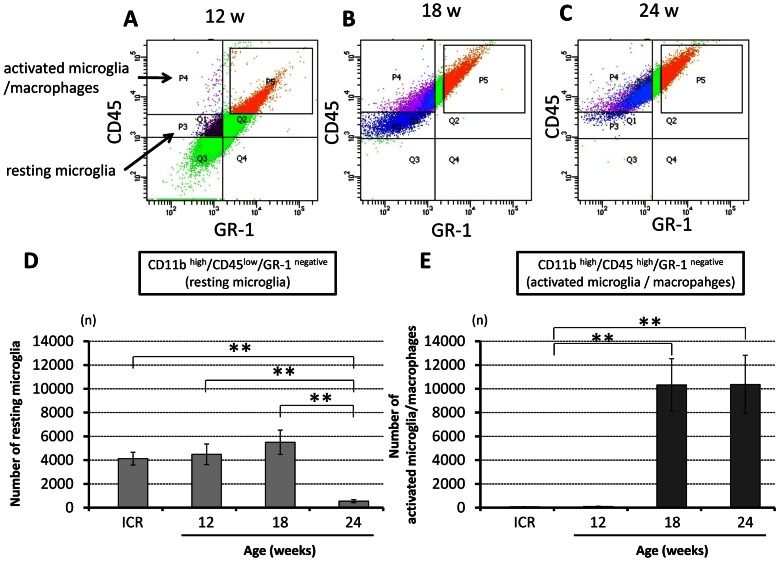
The resting microglia population decreased in association with increased severity of spinal cord compression in *twy/twy* mice. Semi-quantitative flow cytometric analysis of resting microglia and activated microglia/macrophages in the CD11b ^positive^ cells (10.4±0.7% of the spinal cord cells) according to the degree of spinal cord compression. Representative data for 12- (A), 18- (B) and 24-week-old (C) *twy/twy* mice. CD11b^high^ cells in the spinal cord were sub-fractioned into a CD45^low^/GR-1^negative^ population, identifying them as resting microglia; or CD45^high^/GR-1^negative^ population, which identified them as activated microglia/macrophages. The numbers of resting microglia (CD11b^high^/CD45^low^/GR-1^negative^ cells) were significantly lower in 24-week-old mice (D), while the numbers of activated microglia/macrophages (CD11b^high^/CD45 ^high^/GR-1^negative^ cells) were higher in 18- and 24-week-old *twy/twy* mice (E). The number of resting microglia and activated microglia/macrophages in control ICR mice was same as in 12-week-old *twy/twy* mice (D, E). Data are mean±SD. **p*<*0.01 (n = 3 for each time point).

To further determine the activity of CD11b-positive cells in the chronically compressed spinal cords, the profile of the CD11b-positive cells was analyzed by flow cytometry (Fig. 4). Of the 250,000 spinal cord cells, 10.4±0.7% (26,041±1,794 cells) was CD11b ^positive^. The immunoprofile of CD11b-positive cells shifted from CD11b^high^/CD45^low^/GR-1^negative^ cells (resting microglia) to CD11b^high^/CD45 ^high^/GR-1^negative^ cells (activated microglia/macrophages) with the advancement of spinal cord compression (Figs. 4A–C). While almost all CD11b-positive cells were resting microglia in control ICR mice (with the same results seen in each age group; Fig. S2A) and in 12-week *twy*/*twy* mice, the population of activated microglia/macrophages increased significantly in the 18- and 24-week *twy*/*twy* mice (Figs. 4D and E).

**Figure 5:**
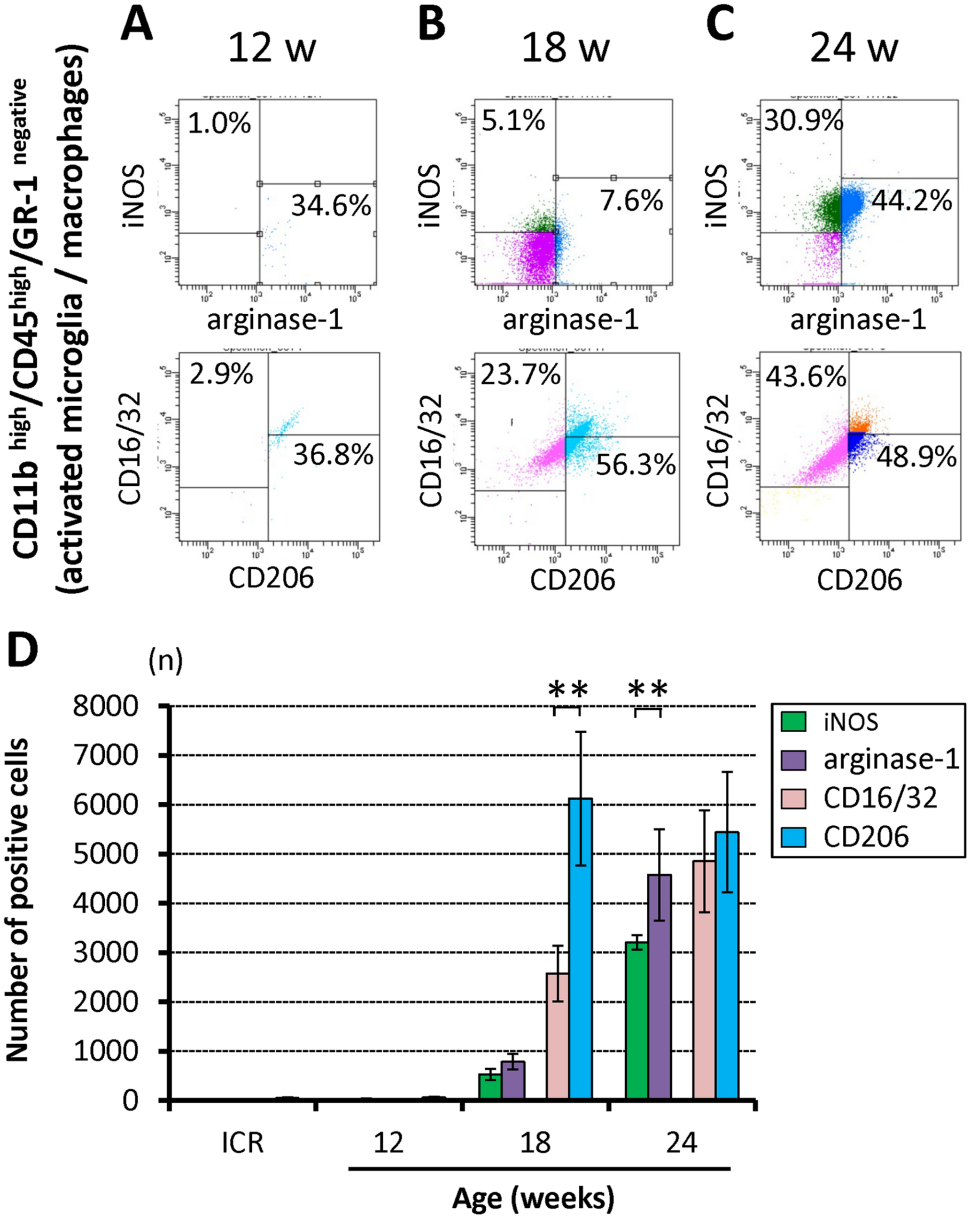
Expression of the M1 phenotype in activated microglia/macrophages correlated to increased severity of spinal cord compression in *twy/twy* mice. Semi-quantitative flow cytometric analysis of iNOS, CD16/32, arginase-1, and CD206 in activated microglia/macrophages. Representative data for 12- (A), 18- (B), and 24- (C) week-old *twy/twy* mice. The number of iNOS ^positive^ and CD16/32 ^positive^ activated microglia/macrophages increased with the worsening of spinal cord compression. Arginase-1 ^positive^ and CD206 ^positive^ cell populations were the predominant in cells present 18- and 24-week-old *twy/twy* mice (D). Data are mean±SD. **p*<*0.01 (n = 3 for each time point).

We also determined the subtypes of resting microglia and activated microglia/macrophages in terms of M1/M2 phenotypic marker expression (Fig. 5). Of the CD11b^high^/CD45 ^low^/GR-1^negative^ cells (resting microglia), 10.2±2.2% (458±97 cells) in 12-week and 73.0±16.1% (4017±884 cells) in 18-week-old *twy*/*twy* mice were arginase-1 ^positive^, while only a few were iNOS^ positive^, CD16/32^ positive^ or CD206^ positive^. The number of arginase-1 ^positive^ resting microglia in samples from control ICR mice of each age group were the same as in 12-week-old *twy/twy* mice (Fig. S3). Of the CD11b^high^/CD45 ^high^/GR-1^negative^ cells (activated microglia/macrophages), 5.1±1.1% (531±112 cells) were iNOS^ positive^, 7.6±1.5% (788±156 cells) were arginase-1 ^positive^, 23.7±5.2% (2577±564 cells) were CD16/32 ^positive^ and 56.3±12.5% (6123±1358 cells) were CD206^ positive^ in the 18 week *twy*/*twy* mice (Fig. 5B). In the activated microglia/macrophages in the 24-week *twy*/*twy* mice, 30.9±1.4% (3205±150 cells), 44.2±8.9% (4578±925 cells), 43.6±9.3% (4851±1032 cells), and 48.9±11.0% (5442±1223 cells) were iNOS^ positive^, arginase-1 ^positive^, CD16/32 ^positive^ and CD206^ positive^, respectively (Fig. 5C). These results indicated that the prevalence of microglia/macrophages of M2 phenotype (arginase-1 and CD206) remained dominant compared to that of the M1 phenotype (iNOS and CD16/32), despite the advancement of spinal cord compression, in agreement with the results of immunostaining. In some of these dot plots, a proportion of microglia/macrophages were double positive to M1 (CD16/32) and M2 (CD206) phenotypic markers (<15%).

### M2 Microglia/Macrophages are a Source of Neurotrophic Factors in the Chronically Compressed Spinal Cord

**Figure 6:**
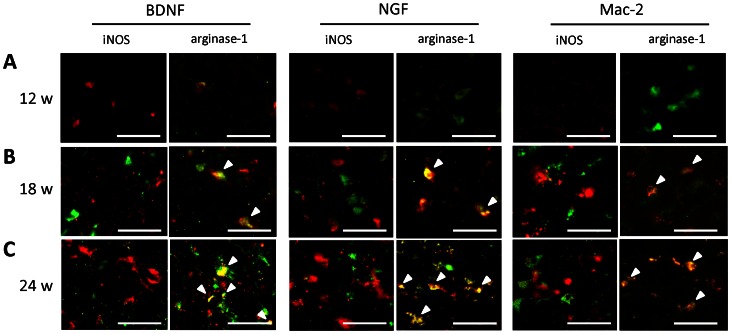
Expression of brain-derived neurotrophic factor (BDNF), nerve growth factor (NGF) and macrophage antigen (Mac)-2 correlated to the phenotype of activated microglia/macrophages and increased spinal cord compression in *twy/twy* mice. Immunofluorescence staining for the expression of BDNF, NGF, and Mac-2 (red) colocalized with iNOS and arginase-1 (green) in the anterior column of 12- (A), 18- (B) and 24-week-old (C) *twy/twy* mice (n = 5 for each time point). The expression levels of neurotrophic factors and Mac-2-positive cells increased with the worsening of spinal cord compression. Neurotrophic factor and Mac-2 colocalized with arginase-1- and CD206-positive cells (arrow heads), but not with iNOS- and CD16/32-positive cells. Scale bars = 50 µm. A–C microphotographs were taken using confocal laser scanning microscope.

To evaluate the expression of neurotrophic factors and phagocytic activity in correlation with M1 and M2 phenotypes, double immunofluorescence staining for BDNF, NGF, and Mac-2 with either iNOS or arginase-1 was performed. Double-positive cells were found particularly in the anterior horn and anterior column. In 12-week *twy*/*twy* mice, some expression of BDNF, NGF and no expression of Mac-2 was found (Fig. 6A): those expressions were also noted in 18-week-old *twy*/*twy* mice (Fig. 6B) and their intensity reached peak levels in 24-week-old *twy*/*twy* mice (Fig. 6C). Some neurotrophic factors and Mac-2 colocalized with arginase-1- or CD206-positive cells, whereas they did not with iNOS- or CD16/32-positive cells (Fig. 6). These results indicate that the M2 phenotype, but not the M1 phenotype, is a source of neurotrophic factors.

### Helper T Cells, but not Neutrophils, Infiltrate the Chronically Compressed Spinal Cord

**Figure 7:**
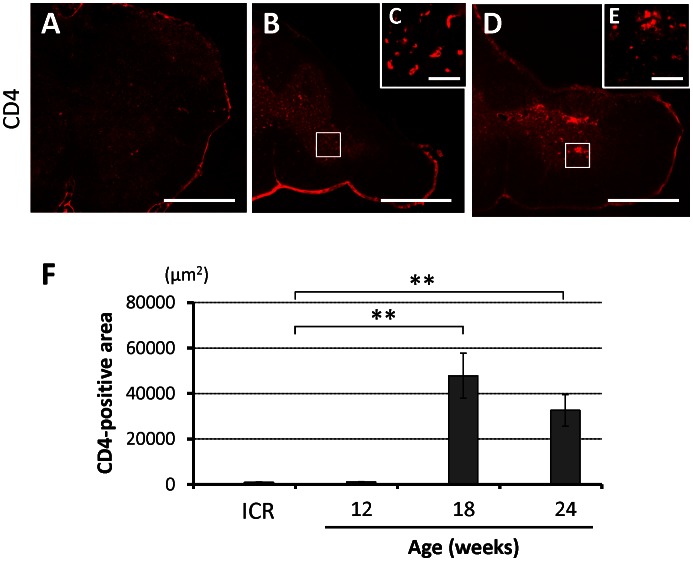
The prevalence of CD4-positive cells in the spinal cord of *twy/twy* mice. Immunostaining of infiltrating helper T cells increased with the worsening of spinal cord compression, especially in 18-week-old *twy*/*twy* mice (A–E). Panel (C) and (E) are high-power photographs of the anterior horn (boxed area). (F) CD4-positive area in control ICR, 12-, 18-, and 24-week-old *twy*/*twy* mice. Scale bar = 500 µm (A, B, D); 200 µm (C, E). Data are mean±SD. **p*<*0.01 (n = 3 for each time point).

To evaluate the infiltration of neutrophils and helper T cells in the chronically compressed spinal cord, tissues were stained for MPO and CD4. No MPO-positive cells and very small amount of MPO activity were detected irrespective of the degree of spinal cord compression (Fig. S4), while the area of CD4-positive increased with advancement of spinal cord compression; especially in the gray matter of 18-week-old *twy*/*twy* mice (Figs. 7A–F). Few positive cells were seen in each age group of the control ICR mice and in 12-week-old *twy*/*twy* mice (Fig. S1C).

### Increased Expression of Th2 Cytokines and Neurotrophic Factors in Chronically Compressed Spinal Cord

**Figure 8:**
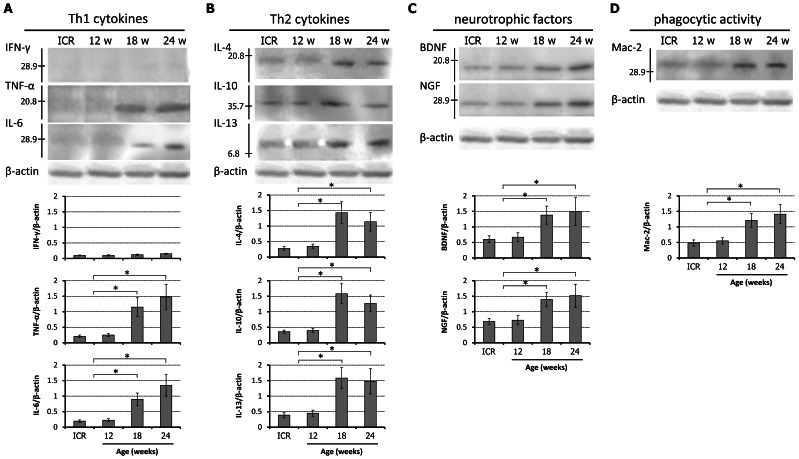
Cytokine expression and phagocytic activity in the *twy/twy* mouse. Immunoblot analysis of T helper 1 (Th1), T helper 2 (Th2) cytokines, neurotrophic factors and phagocytic activity in control ICR, 12, 18, and 24-week-old *twy/twy* mice. (A) The expression of IFN-γ was not detected in all age groups, but the expression levels of TNF-α and IL-6 increased significantly with the severity of spinal cord compression. (B) The expression levels of Th2 cytokines were highest in 18-week-old and remained significantly elevated in 24-week-old *twy/twy* mice. (C) The expression levels of neurotrophic factors and Mac-2 (D) increased significantly with the severity of spinal cord compression. Each graph shows the relative band intensity normalized to that of β-actin. Data are mean±SD. *p*<*0.05 (n = 3 for each time point).

Western blotting was performed to evaluate the correlation between the severity of spinal cord compression and IFN-γ, TNF-α, IL-6, IL-4, IL-10, IL-13, BDNF, NGF, and Mac-2 protein levels (Fig. 8). IFN-γ (28 kDa band) was weakly expressed and its expression level did not change with age. The intensities of the bands of TNF-α (19 kDa band) and IL-6 (26 kDa band) increased with worsening of spinal cord compression (Fig. 8A). The intensities of the bands for IL-4 (18 kDa band), IL-10 (37 kDa band), and IL-13 (13 kDa band) reached peak levels in 18-week *twy*/*twy* mice, but somewhat decreased in 24-week *twy*/*twy* mice (Fig. 8B). The expression of these proteins in control ICR mice (with the same results seen in samples of different age groups; Fig. S2B) was the same as that seen in 12-week *twy*/*twy* mice. These results indicate increased Th1 cytokine expression reflects worsening of spinal cord compression, whereas increased Th2 cytokine expression seems to reflect changes in the population of activated microglia. On the other hand, the intensities of the bands for BDNF and NGF, as well as Mac-2, increased significantly with advancement of spinal cord compression (Figs. 8C and D).

## Discussion

We reported in the present study that chronic progressive compression of the cervical spinal cord due to a calcified lesion of the atlantoaxial membrane at C1–C2 in the *twy/twy* mouse caused neuronal loss, as indicated by NeuN-positive cells, an increase in CD11b-positive cells in both the grey matter and white matter, and infiltration of CD 4-positive cells in the grey matter as well as few MPO positive cells and the activity, with these changes seen at the site of the compression. The following are the main findings: 1) the number of CD11b positive microglia/macrophages increased with the severity of spinal cord compression; 2) there was a shift in the polarity of M1 and M2 cells present, with the prevalence of M1 cells increasing with the severity of spinal cord compression; 3) the number of resting microglia decreased in proportion with increases in activated microglia/macrophages; 4) arginase-1-positive cells, but not iNOS-positive cells, expressed neurotrophic factors (BDNF, NGF) and higher Mac-2 immunoreactivity; 5) an increase in Th1 cytokines increased with the severity of spinal cord compression, while an increase in Th2 cytokines was observed concurrently with changes in presence of the activated microglia population.

Several groups have characterized the pathological features of chronic spinal cord compression of patients with CSM and OPLL [5]–[7] or of the animal model of spinal cord compression seen in the *twy/twy* mouse [8], [9], [33]–[37]. Significant similarities in histopathological and pathophysiological changes have been described in chronic progressive spinal cord compression and traumatic SCI. These similarities include a decrease in the neuronal population with degeneration and demyelination followed by a cascade of “secondary” injury [2]. Activated microglia, derived from resting microglia, and recruited macrophages from the peripheral circulation are among the main effector cells of the inflammatory response that follows SCI, and are associated with the production of proinflammatory cytokines and related immune effector molecules that can induce both necrotic and programmed cell death, which correlate with neurological deficit [10], [11], [16]. Activation of microglia after SCI is evident on day one, and the number of activated microglia has been shown to increase during the first 7 days and then plateau, 2–4 weeks after injury; with small differences related to the animal strain being used as a model [13]. The present results demonstrated a decrease in the NeuN-positive area in the anterior horn opposite the posterior compression and posterior horn and an increase in the CD11b-positive area, especially in the anterior horn and anterior column; these changes were in proportion with the degree of spinal cord compression. In our previous studies, we observed a significant reduction in the number of remaining surviving neurons when the transverse remnant area of the spinal cord of the *twy/twy* mouse spinal cord decreased to ≤70% of the control (*+/twy* mice) [33], and further that the extent of demyelination and Wallerian degeneration in the white matter increased proportionately with the magnitude of spinal cord compression [35]. We also reported a significant increase in TUNEL-positive cells in the anterior horn and also glial cells in the white matter, in spinal cord segments located rostral and caudal to the segment with maximal compression [8]. It is well-known that activated microglia/macrophages secrete cytotoxic substances including various cytokines (e.g., TNF-α, IL-1 and -6), reactive free radicals, and nitric oxide. In addition, the major role of microglia at the lesion centre is probably rapid phagocytosis of debris [25], [40]. Our results suggest that neuronal loss through the mechanisms of necrosis and apoptosis is associated with activation of microglia/macrophages, production of proinflammatory cytokines by these cells, and putative phagocytosis, which may also be associated with axonal demyelination in the *twy/twy* spinal cord. This chain of events is somewhat similar to the delayed apoptosis of oligodendrocytes following activation of microglia after SCI [41].

As part of the response to chronic and progressive spinal cord compression, the present results showed high numbers of CD11b-positive cells, representing resting and activated microglia, as well as recruited macrophages, at different stages of microglia/macrophages activation and polarization [29]. The proportion of both M1 and M2 microglia/macrophages increased with the severity of spinal cord compression with abundant cells appearing as the M2 phenotype at 24 weeks in *twy/twy* mice. Furthermore, the results also showed increased M1/M2 antigen expression rate, a ratio known to correlate with CNS damage and repair [22]. M2 microglia/macrophages seem to promote neuroprotection, neurogenesis, and repair even in severely compressed spinal cord. On the other hand, further progression of the spinal cord compression may have resulted in the induction of CD11b -positive cells into the M1 phenotype, which is known for its deleterious effects [27]. We reported previously the presence of axonal and dendritic outgrowth, which were induced by overexpression of neurotrophic factors at the site of compression in the *twy/twy* mice spinal cord [36]. Immunostaining in the present study demonstrated high co-expression of neurotrophic factors in M2 phenotype macrophages as well as increased expression of Mac-2, which reflects enhanced phagocytic activity [40], [42]. These changes possibly represent the spinal cord response to chronic progressive spinal cord compression in order to maintain neuronal function [28], [36].

The results of flow cytometry provided further details on the phenotypes of the involved cells, where resting microglia mainly expressed M2 markers, as described previously by other groups [17], [32]. Our data confirmed that resting microglia are also the source of activated microglia/macrophages, since the population of resident microglia decreased while that of the activated microglia/macrophages increased in response to severity of spinal mechanical compression. In addition, the phenotypic changes in activated microglia/macrophages showed dynamic behavior with important M2 phenotype populations in 18- and 24-week-old *twy/twy* mice and further increase in the M1 phenotype at 24 weeks, probably reflecting worsening of chronic spinal cord compression. The results related to the serial changes in the expression levels of phenotypic markers in activated microglia deserve special attention. It has already been documented both *in vitro* and *in vivo* that resting microglia possesses the M2 phenotype and are the only neural cells that express arginase-1 [26], [32]. However, after their activation, these cells express different markers in a time-related manner. For example, arginase-1 is a well-documented early marker expressed in those cells that undergo the alternative pathway of activation to acquire the M2 macrophage phenotype, whereas iNOS is a marker of cells activated through the classical pathway, changing into M1 activated macrophages [22]. As the activation process advances, the fully mature activated cells express CD16/32 and CD206, which are phenotypic hallmarks of M1 and M2 macrophages, respectively [17], [28]. Our immunohistochemical and flow cytometric data showed a similar behavior where the expression of such phenotypic markers increased with time, representing completion of the macrophage activation process. CD206 is an endocytic receptor that recognizes glycoprotein for antigen processing and presentation and is widely used to identify the M2 phenotype [26]. Our results showed that the expression of iNOS and arginase-1 persisted and increased with time; possibly related to the persistence of the insult triggering microglial/macrophages activation, since these markers mainly reflect the early stages of activation [22].

Cells that were double immunopositive for M1 and M2 phenotypic markers were seen locally within the spinal cord, which is highly suggestive of microglia/macrophages undergoing phenotype change due to environmental influences created by the cervical compression. Polarization of the microglia/macrophages requires an active response in the form of inflammatory cytokines, other immune effector molecules and neurotrophic factors released from the cells in the injured tissue as well as from different cells recruited to the site of the primary insult [27]. The sources of these cytokines remain controversial. Astrocytes seem to be the main source of TNF-α since they are the most abundant cell type after SCI capable of producing this cytokine [43]. Previous studies indicated that the main source of IFN-γ is blood-derived cells recruited to the site of injury (macrophages, natural killer cells, neutrophils and helper T cells) through the inflammatory process mediated by some ILs [44], [45]. Some of these recruited cells can produce Th2 cytokines, but the main source of IL-4 and IL-13 seems to be microglia [46], [47]. However, the pathophysiology of chronic spinal cord compression in the *twy/twy* mouse spinal cord could include a limited blood cell access into the injured spinal cord, and there is no evidence of the destruction of the blood spinal cord barrier [33], [35], [37], thus limiting the infiltration of helper T cells and neutrophils. Under such circumstances, the expression of IFN-γ is expected to be weak and TNF-α becomes the most important Th1 cytokine responsible for macrophage activation. The preserved blood spinal cord barrier would also limit the supply of Th2 cytokines; leaving resting and activated microglia at the injury site as the main source of Th2 cytokines for alternative activation of microglia/macrophages [46], [47]. While there is no evidence to our knowledge of disruption of the blood spinal cord barrier in the *twy*/*twy* mouse to date, future research may prove otherwise. For analysis of this aspect, special *twy/twy* background chimeric mice models would be required [42], which are not currently commercially available; therefore, the possibility of cytokines deriving from blood and having an important role in determining inflammatory and immunomodulatory activity within the spinal cord remains as a possible limitation of our animal model. Our immunoblot results provided insight on the polarization process; the expression of Th2 cytokines increased from 18 weeks in possible correlation with the enlarged activated microglia population. The transient increase of Th2 cytokines explains our findings of M2 phenotype being the predominant type of activated cell; which would also explain the enhanced expression of neurotrophic factors and Mac-2 [19], [26], [29]. The present results also demonstrated increased expression of Th1 cytokines, as reported previously by our group [8], together with the reported increase in IL-6 expression as part of the inflammatory process [42], [48]. Thus, over-expression of TNF-α in the *twy/twy* mice may be the most important Th1 cytokine responsible for M1 macrophage activation [28].

Interestingly, the resultant increase in the neurotropic factors at the site of spinal cord compression did not reverse the pathological changes of increased expression of proinflammatory cytokines and apoptosis of neurons and glia. Accordingly, the only treatment available at present is the surgical removal of the cause of chronic spinal cord compression [1]–[4]. However, surgery is unlikely to reverse the symptoms in patients with advanced cervical compressive myelopathy. In such patients, the use of specific antibodies against Fas ligand to reduce apoptosis of neurons and oligodendrocytes [7], [37], promotion of neuronal regeneration, through blockade of the inflammatory cascade, and induction of the M2 phenotype [48] are promising options for combined medical therapies in future management of this condition.

In conclusion, the present study demonstrated that chronic and progressive spinal compression induced over-expression of Th1 cytokines (TNF-α, IL-6) and increased the population of classically activated macrophages (M1 phenotype). These changes could be responsible, at least in part, for neuronal loss and may also induce the demyelination of axons found in CSM and OPLL; however, they could also induce alternative activation by Th2 cytokines (IL-4, -10, and -13) and increase M2 microglia/macrophages, which provide neuroprotection and enhanced phagocytic activity. This work provides the rationale for therapeutic targeting of alternative activation of microglia/macrophages in human CSM and OPLL.

## Supporting Information

Figure S1 **Assessment of aging effects in control ICR mice by immunofluorescent staining.** (A) Immunofluorescence staining for the expression of CD11b (red) and NeuN (green) in 12-, 18- and 24-week-old ICR mice. (B) Immunofluorescence staining for the expression of iNOS and CD16/32 (green) identifying classically activated microglia/macrophages (M1 phenotype) and arginase-1 and CD206 (green) characterizing alternatively activated microglia/macrophages (M2 phenotype); co-localized with CD11b (red) in the anterior column of 12-, 18- and 24-week-old ICR mice. (C) Immunostaining of infiltrating helper T cells in 12-, 18- and 24-week-old ICR mice. In these assessments, there were no differences between samples from different ages. Scale bars = 500 µm (A-upper row, C), 50 µm (A-lower row, B).
(TIF)

Figure S2 **Assessment of aging effect in control ICR mice by flow cytometry and immunoblot analysis.** (A) Semi-quantitative flow cytometric analysis of resting microglia and activated microglia/macrophages in the CD11b ^positive^ cells in 12-, 18- and 24-week-old ICR mice. (B) Immunoblot analysis of T helper 1 (Th1), T helper 2 (Th2) cytokines, neurotrophic factors and phagocytic activity in 12-, 18- and 24-week-old ICR mice. In these assessments, there were no differences between samples from different ages.
(TIFF)

Figure S3 **Characterization of resting microglia population in spinal cord of** ***twy/twy*** **mice.** Semi-quantitative analysis for iNOS, CD16/32, arginase-1, and CD206 in resting microglia was performed in flow cytometry. Representative data of 12- (A), 18- (B), and 24- (C) week-old *twy/twy* mice (n = 3 for each time point). Arginase-1 ^positive^ resting microglia constituted 10.2±2.2% (458±97 cells) and 73.0±16.1% (4017±884 cells) of the cells in 12- and 18-week-old mice. The number of arginase-1 ^positive^ resting microglia in control ICR mice was same as in 12-week-old *twy/twy* mice. Only a few iNOS-, CD16/32- and CD206-positive resting microglia were present in control ICR and *twy/twy* mice (D). Data are mean±SD.
(TIF)

Figure S4 **Assessment of the neutrophil populations and myeloperoxidase (MPO) activity in the spinal cord of** ***twy/twy*** **mice.** To examine the presence of neutrophils in the compressed spinal cord, we examined the presence of MPO by immunohistochemistry and by assay for MPO activity. MPO immunostaining demonstrated the lack of infiltration of neutrophils (A–C), whereas biochemical assay indicated a very small amount of MPO activity (D) irrespective of the severity of the spinal cord compression (n = 3 for each time point). Scale bars = 500 µm. Data are mean±SD.
(TIF)

Table S1 This table shows experimental groups used in the present study.
(DOCX)
