# Supplementary figures and images for: The Prevalence and Phenotype of Activated Microglia/Macrophages within the Spinal Cord of the Hyperostotic Mouse (twy/twy) Changes in Response to Chronic Progressive Spinal Cord Compression: Implications for Human Cervical Compressive Myelopathy
Source: PLoS One. 2013 May 24;8(5):e64528. doi: 10.1371/journal.pone.0064528 (PMC3663759; doi:10.1371/journal.pone.0064528)

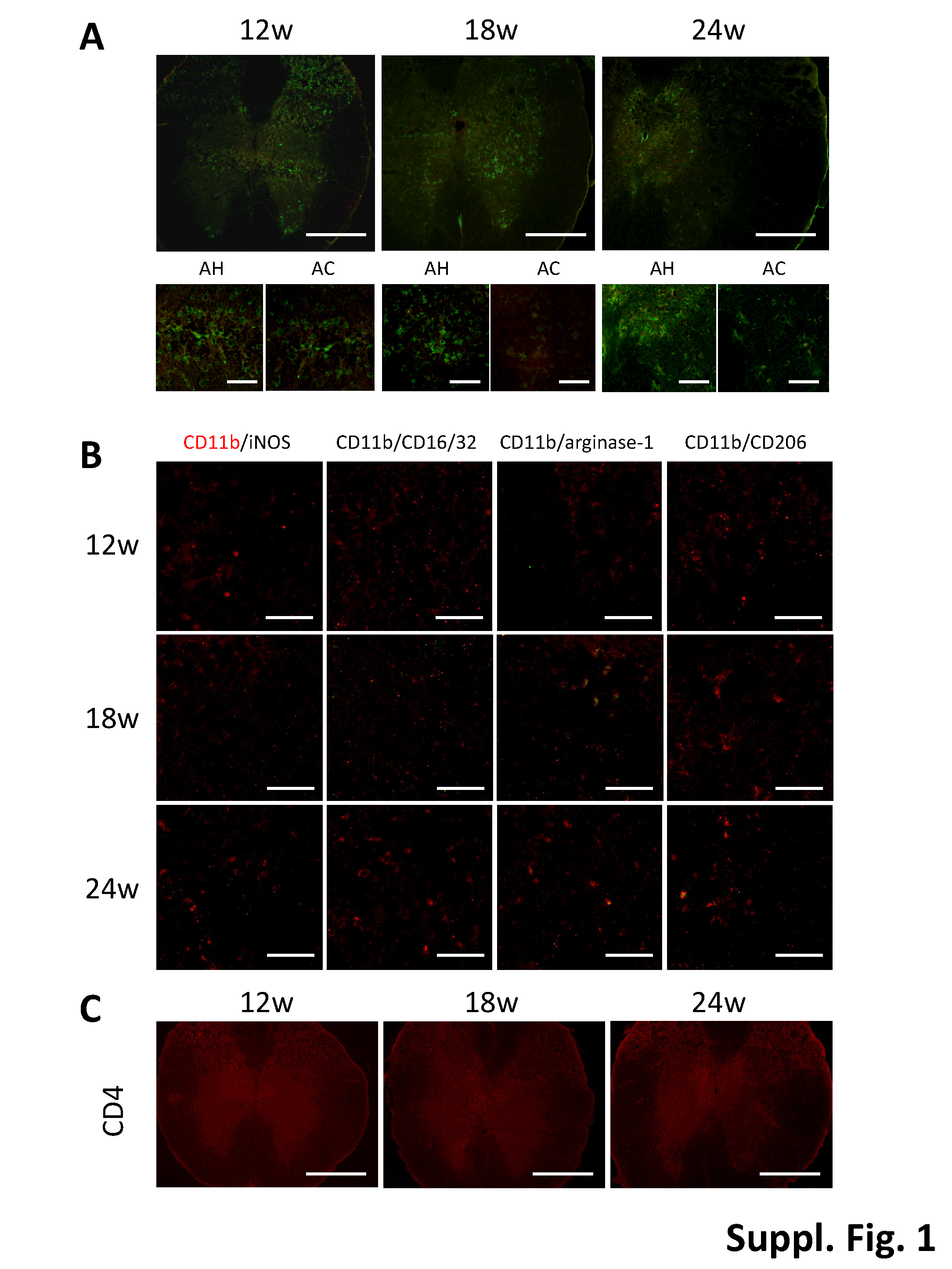

Supplement: Figure S1 — Assessment of aging effects in control ICR mice by immunofluorescent staining. (A) Immunofluorescence staining for the expression of CD11b (red) and NeuN (green) in 12-, 18- and 24-week-old ICR mice. (B) Immunofluorescence staining for the expression of iNOS and CD16/32 (green) identifying classically activated microglia/macrophages (M1 phenotype) and arginase-1 and CD206 (green) characterizing alternatively activated microglia/macrophages (M2 phenotype); co-localized with CD11b (red) in the anterior column of 12-, 18- and 24-week-old ICR mice. (C) Immunostaining of infiltrating helper T cells in 12-, 18- and 24-week-old ICR mice. In these assessments, there were no differences between samples from different ages. Scale bars = 500 µm (A-upper row, C), 50 µm (A-lower row, B). (TIF) [file pone.0064528.s001.tif]

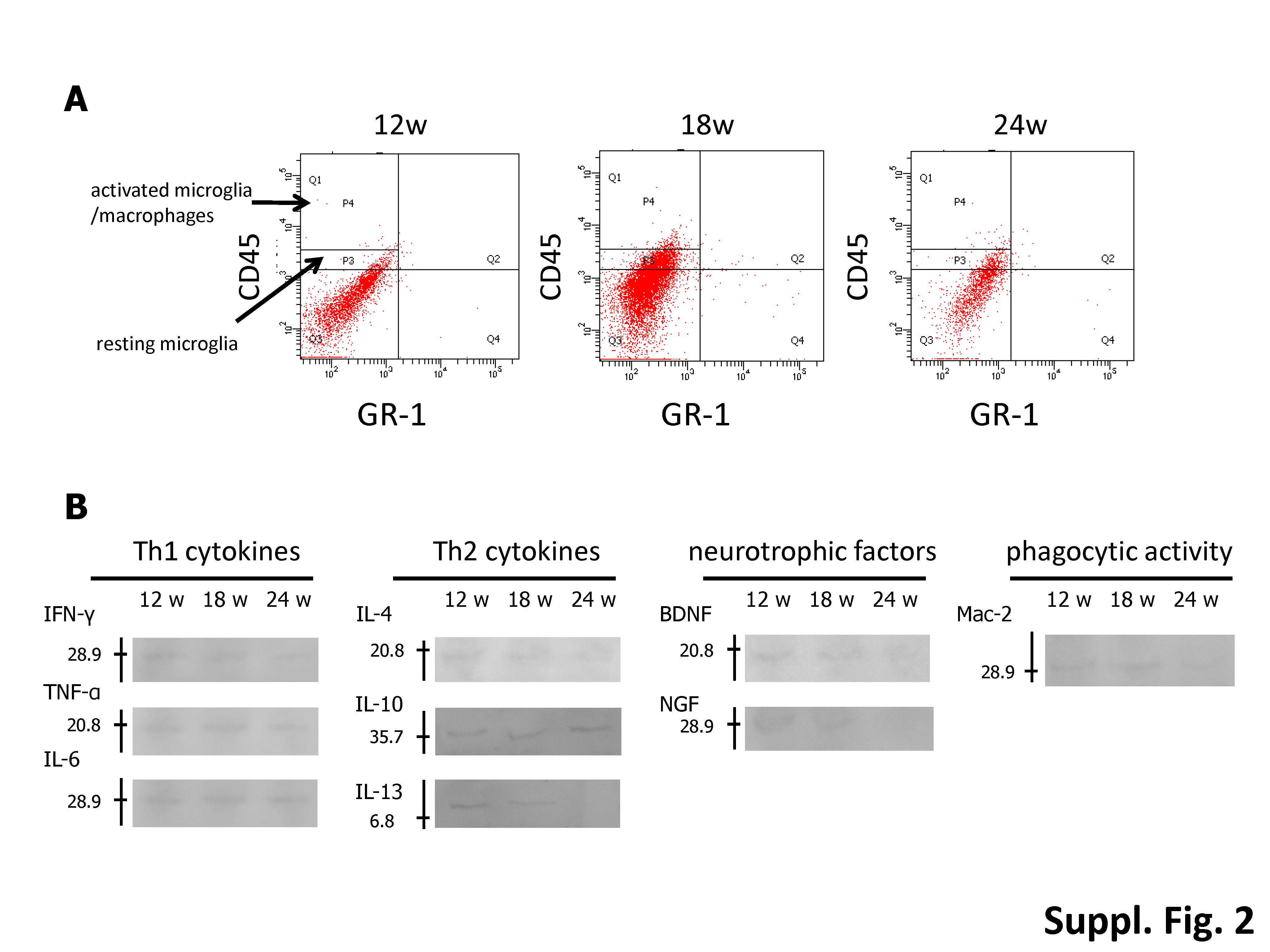

Supplement: Figure S2 — Assessment of aging effect in control ICR mice by flow cytometry and immunoblot analysis. (A) Semi-quantitative flow cytometric analysis of resting microglia and activated microglia/macrophages in the CD11b positive cells in 12-, 18- and 24-week-old ICR mice. (B) Immunoblot analysis of T helper 1 (Th1), T helper 2 (Th2) cytokines, neurotrophic factors and phagocytic activity in 12-, 18- and 24-week-old ICR mice. In these assessments, there were no differences between samples from different ages. (TIFF) [file pone.0064528.s002.tiff]

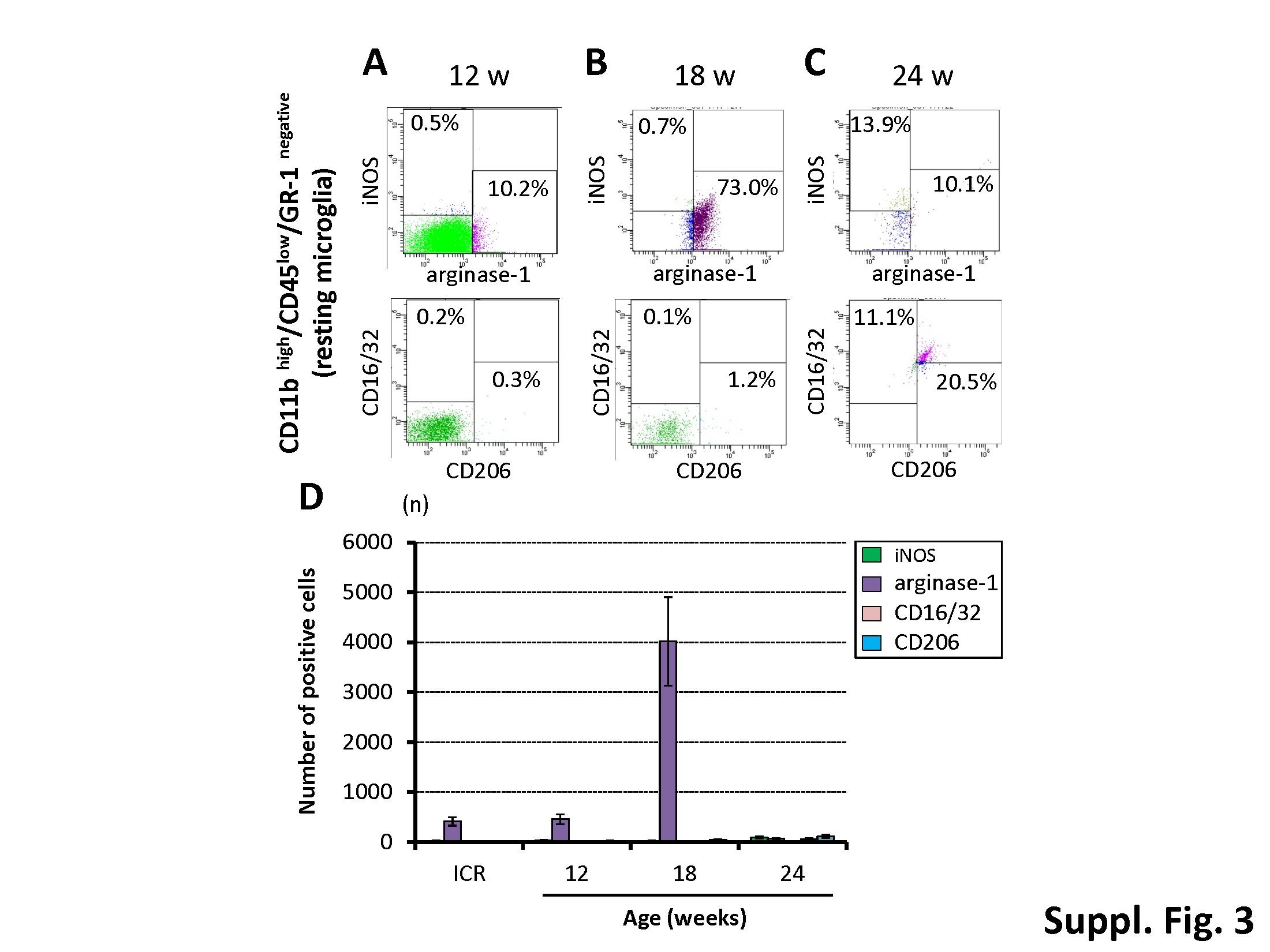

Supplement: Figure S3 — Characterization of resting microglia population in spinal cord of twy/twy mice. Semi-quantitative analysis for iNOS, CD16/32, arginase-1, and CD206 in resting microglia was performed in flow cytometry. Representative data of 12- (A), 18- (B), and 24- (C) week-old twy/twy mice (n = 3 for each time point). Arginase-1 positive resting microglia constituted 10.2±2.2% (458±97 cells) and 73.0±16.1% (4017±884 cells) of the cells in 12- and 18-week-old mice. The number of arginase-1 positive resting microglia in control ICR mice was same as in 12-week-old twy/twy mice. Only a few iNOS-, CD16/32- and CD206-positive resting microglia were present in control ICR and twy/twy mice (D). Data are mean±SD. (TIF) [file pone.0064528.s003.tif]

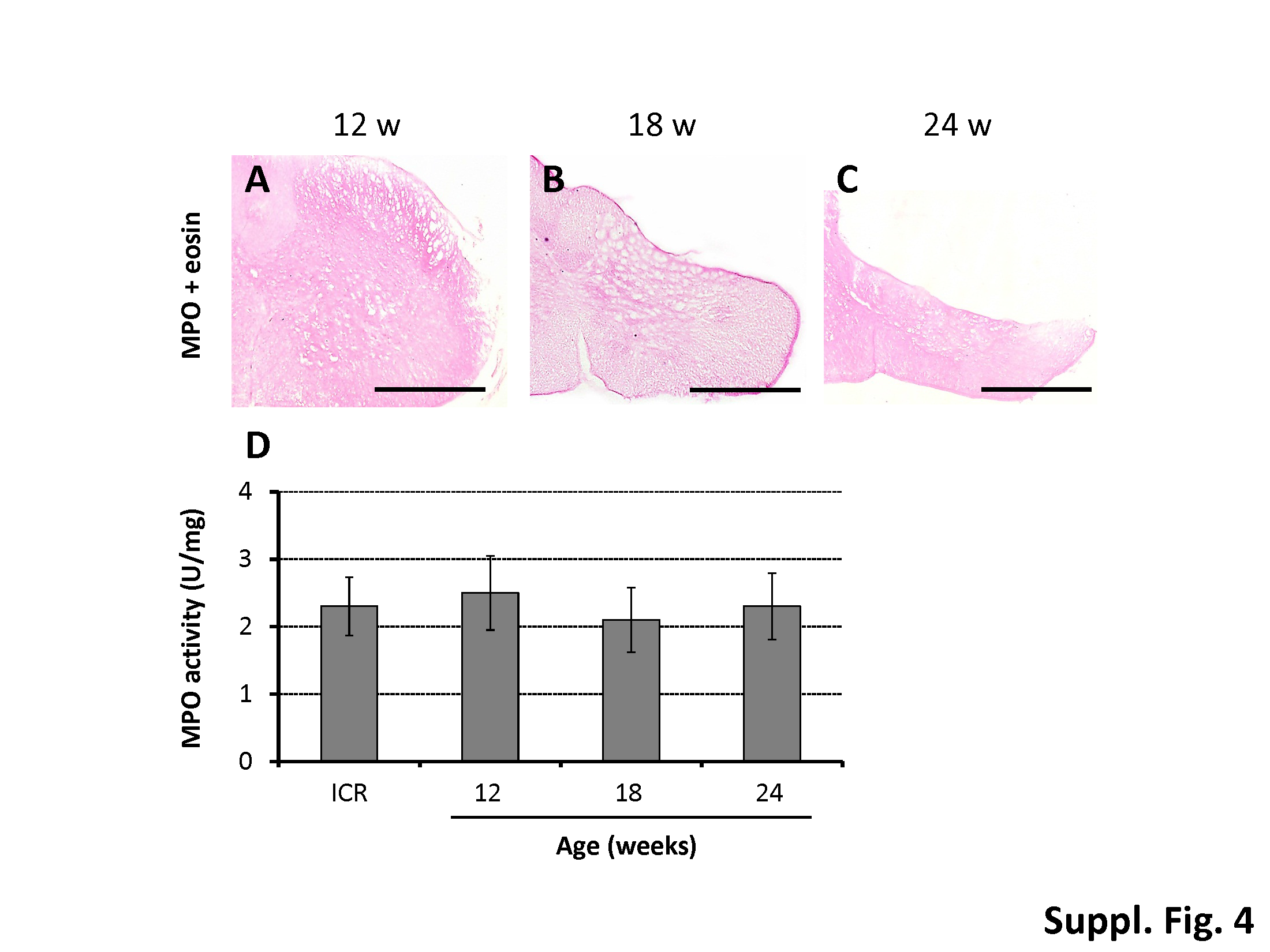

Supplement: Figure S4 — Assessment of the neutrophil populations and myeloperoxidase (MPO) activity in the spinal cord of twy/twy mice. To examine the presence of neutrophils in the compressed spinal cord, we examined the presence of MPO by immunohistochemistry and by assay for MPO activity. MPO immunostaining demonstrated the lack of infiltration of neutrophils (A–C), whereas biochemical assay indicated a very small amount of MPO activity (D) irrespective of the severity of the spinal cord compression (n = 3 for each time point). Scale bars = 500 µm. Data are mean±SD. (TIF) [file pone.0064528.s004.tif]
