# Supplementary material for: The Prevalence and Phenotype of Activated Microglia/Macrophages within the Spinal Cord of the Hyperostotic Mouse (twy/twy) Changes in Response to Chronic Progressive Spinal Cord Compression: Implications for Human Cervical Compressive Myelopathy
Source: PLoS One. 2013 May 24;8(5):e64528. doi: 10.1371/journal.pone.0064528 (PMC3663759; doi:10.1371/journal.pone.0064528)
Supplement: Table S1 — This table shows experimental groups used in the present study. (DOCX) [file pone.0064528.s005.docx]

**Table S1. Experimental groups used in the present study.**

|  | ICR: Age (weeks) | | | *twy*/*twy*: Age (weeks) | | |
| --- | --- | --- | --- | --- | --- | --- |
|  | 12 | 18 | 24 | 12 | 18 | 24 |
| *Gross morphology (H&E staining) (Figure 1) |  |  |  |  |  |  |
| axial section |  |  |  | 3 | 3 | 3 |
| sagittal section |  |  |  | 2 | 2 | 2 |
| Immunostaining for CD11b/Nissl, iNOS, CD16/32, arginase-1, CD206;  5 axial and 2 sagittal sections in each *twy*/*twy* mouse (Figures 2 and 3, Figure S1) | 2 | 2 | 2 | 7 | 7 | 7 |
| *Flow cytometry (Figures 4, 5, Figure S2, 3) | 2 | 2 | 2 | 3 | 3 | 3 |
| Immunostaining for BDNF, NGF, Mac-2/iNOS, arginase-1 (Figure 6) |  |  |  | 5 | 5 | 5 |
| Myeloperoxidase (MPO) staining, immunostaining for CD4 (Figure 7, Figure S1, 4) | 2 | 2 | 2 | 3 | 3 | 3 |
| MPO activity | 2 | 2 | 2 | 3 | 3 | 3 |
| *Immunoblot analysis (Figure 8, Figure S2) | 2 | 2 | 2 | 3 | 3 | 3 |

H&E=hematoxylin-eosin; iNOS=inducible nitric oxide synthase; BDNF=brain-derived neurotrophic factor; NGF=nerve growth factor; Mac-2=macrophage antigen-2.

*Computed tomography (CT) scan was obtained before each experiment.
